# UTY (KDM6C) in Cancer: Epigenetic Regulation, Tumour Suppressor Functions, and Clinical Implications

**DOI:** 10.3390/epigenomes10020031

**Published:** 2026-05-09

**Authors:** Chitrakshi Chopra, Chandra Prakash Prasad, Manish Kumar

**Affiliations:** 1 Department of Biochemistry, All India Institute of Medical Sciences, Vijaypur, Jammu 181134, India; chitrakshi288@gmail.com; 2 Department of Medical Oncology (Laboratory), Dr. Bhim Rao Ambedkar Institute Rotary Cancer Hospital, All India Institute of Medical Sciences, New Delhi 110029, India; researchchandra@gmail.com

**Keywords:** UTY, UTX, KDM6C, H3K27me3, LOY (loss of Y chromosome), cancer

## Abstract

The ubiquitously transcribed tetratricopeptide repeat Y-linked gene (UTY/KDM6C), a catalytically impaired histone demethylase encoded on the Y chromosome, has garnered increasing attention for its emerging roles in tumorigenesis and cancer progression. Despite high sequence homology with its X-linked paralog UTX/KDM6A, UTY exhibits markedly reduced or absent H3K27me3 demethylase activity due to critical amino acid substitutions in its Jumonji C domain. Consequently, UTY primarily functions through non-enzymatic mechanisms, acting as a scaffold in chromatin-remodelling complexes like COMPASS and SWI/SNF, or mediating protein–protein interactions that regulate transcriptional programs independent of demethylation. This aligns with epigenetic dysregulation in cancers, where imbalances in repressive H3K27me3 and active H3K4me either drive tumour suppressor silencing or oncogene activation. Unlike frequently mutated UTX in cancers such as breast, renal cell carcinoma, and acute myeloid leukaemia, UTY’s contributions in cancer are less defined, constrained by male-specific expression. Emerging evidence suggests UTY as a context-dependent tumour suppressor in AML and squamous-like pancreatic ductal adenocarcinoma. While direct functional validation remains limited in several cancer types, UTY is increasingly implicated as a potential tumour suppressor in haematological malignancies and prostate cancer. Therapeutically targeting UTY’s scaffold functions shows promise for male-specific cancers and merits future investigation.

## 1. Introduction

**Figure 1:**
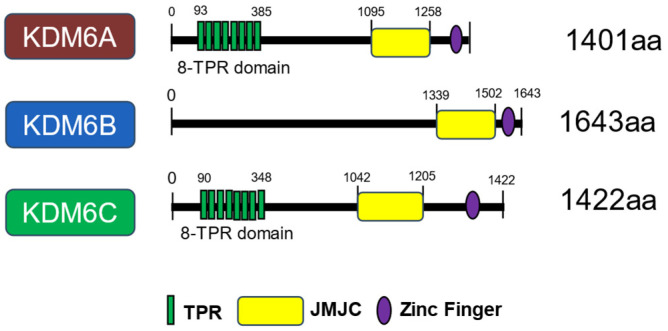
Schematic representation of the protein domains of KDM6 enzymes. The structure of KDM6 members consists of three major regions based on the Uniport Database: tetratricopeptide repeat domain (TPR), Jumonji C (JmjC) domain and zinc finger domain with respective numbers indicate the amino acid residues.

The Y-chromosome gene UTY (also called KDM6C), a ubiquitously expressed tetratricopeptide repeat protein belonging to the KDM6 family of histone demethylases, is a catalytically impaired histone demethylase with minimal H3K27me3 demethylase activity, and has recently drawn interest for its role in tumor development and cancer advancement. Although it shares strong sequence homology with its X-chromosome counterpart UTX (KDM6A), UTY displays markedly diminished or absent demethylase activity, stemming from key amino acid changes in its catalytic Jumonji C domain (JmjC) [1,2]. Consequently, UTY is thought to primarily function through non-catalytic mechanisms, such as serving as a structural component within chromatin-modifying complexes or mediating protein–protein interactions that influence gene expression programs independent of its demethylase function [3,4]. This aligns with broader observations in the field of epigenetics, where dysregulation of histone methylation marks such as the repressive H3K27me3 or active H3K4me2 modifications can lead to the aberrant silencing of tumor suppressor genes or the activation of oncogenes [5]. Recent analyses of public cancer databases have revealed that the expression levels of epigenetic modifiers, including those responsible for histone methylation, are frequently up- or downregulated in cancer cells, suggesting their significant involvement in oncogenic processes [6]. UTY, the Y-linked homolog of UTX, shares structural similarities with its paralog but possesses minimal demethylase activity due to a mutation in the JmjC catalytic domain, leading to questions regarding its functional capacity in vivo [7,8,9]. UTY shares extensive sequence homology with UTX, reaching 88% identity both within and outside the catalytic domain, despite its lack of enzymatic function [10]. This high degree of conservation suggests that UTY retains the structural architecture necessary for protein–protein interactions, allowing it to integrate into chromatin-modifying complexes such as the KDM6 complex [11]. The tetratricopeptide repeat domains present in both proteins further facilitate these interactions by mediating the assembly of multiprotein complexes involved in transcriptional regulation and chromatin remodelling [12]. While the JmjC domain of UTY contains critical amino acid substitutions that abrogate its ability to demethylate H3K27me2 and H3K27me3, the preserved N-terminal tetratricopeptide repeat motifs enable the protein to function as a molecular scaffold that stabilizes the interaction between chromatin modifiers and target gene loci [13,14,15]. The KDM6 subfamily, which includes UTY, is characterized by a multi-domain architecture consisting of tetratricopeptide repeat, helical, linker, JmjC, and GATA-like zinc finger domains that facilitate its interaction with chromatin and response to cellular metabolites [16] (Figure 1). Despite this catalytic deficiency, emerging evidence suggests that UTY retains biological relevance through its incorporation into multiprotein complexes, such as the COMPASS and SWI/SNF assemblies, where it may contribute to the regulation of chromatin accessibility and transcriptional elongation independent of demethylation [17,18]. Consequently, this scaffolding capability allows UTY to participate in the recruitment of co-activators such as P300 and components of the SWI/SNF chromatin remodelling complex, thereby facilitating the activation of developmental regulators through the promotion of open chromatin structures [19,20]. These structural interactions are further mediated by tetratricopeptide repeat domains, which facilitate the assembly of multiprotein complexes involved in transcriptional regulation independent of demethylase activity [21]. This non-enzymatic mode of action is particularly relevant in the context of tumour suppression, where UTY has been identified as a critical regulator of enhancer activity and gene activation through the coordination of COMPASS-mediated H3K4 mono-methylation and CREBBP/p300-mediated H3K27Ac deposition [22]. This functional redundancy is supported by in vivo evidence demonstrating that male *Utx* knockout embryos, which express normal levels of *Uty*, survive until birth, whereas homozygous *Utx* knockout is embryonically lethal in females, indicating that UTY can compensate for UTX loss through demethylation-independent mechanisms [10,23]. Furthermore, the tumor suppressor capacity of UTY appears to be attenuated compared to UTX, as evidenced by its weaker ability to inhibit proliferation in leukaemia models and its lower mutation frequency across various cancer types [9,24].

## 2. Role of UTY in Cancer

### 2.1. Prostate Cancer

**Figure 2:**
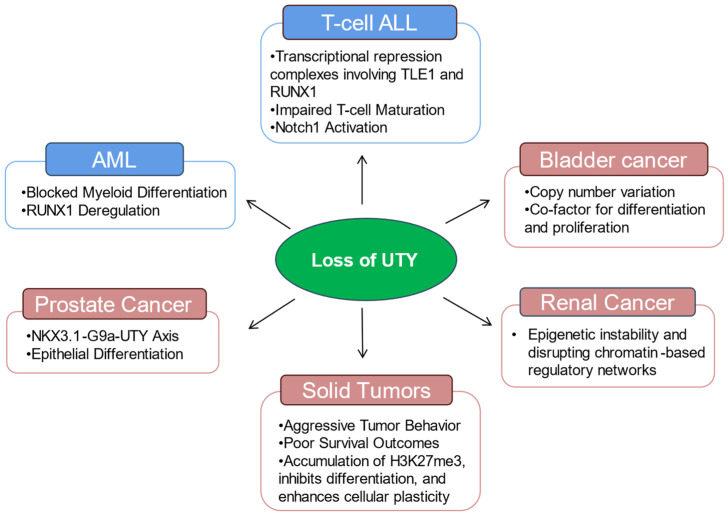
Loss of UTY in different cancers and associated molecular changes. AML: Acute Myeloid Leukemia; T-cell ALL: T-cell acute lymphoblastic Leukemia.

In the context of prostate malignancy, UTY has been observed to exhibit distinct expression patterns that may correlate with disease progression and androgen receptor signalling, although its precise functional contribution remains less defined than that of its paralog UTX. Nevertheless, preliminary investigations suggest that UTY may modulate androgen-responsive gene networks, potentially influencing the transcriptional landscape of prostate tumours through its interaction with chromatin-remodelling machinery [25]. Pan-cancer analyses reveal epigenetic modifier dysregulation and suggest that UTY may influence sex-dimorphic outcomes [3,6]. Therapeutically, targeting UTY’s scaffold functions holds promise for male-specific cancers, including prostate cancer, and warrants further exploration [9,26]. Specifically, UTY has been identified as a component of the KDM6 complex that associates with the androgen receptor, where it may facilitate the recruitment of additional co-activators to target gene promoters despite the lack of catalytic function [25]. This structural role implies that UTY may contribute to the maintenance of androgen-dependent transcriptional programs, potentially affecting tumor growth and therapeutic response in prostate cancer [27]. Functional studies suggest that UTY may contribute to the differentiation of prostate epithelial cells and may be linked to the susceptibility to prostate cancer, functioning as a downstream effector of the transcription factor that is specific to the prostate lineage [28]. NKX3.1 is one of the first genes to be regulated in the developing prostate epithelium. Its expression is often diminished during the early stages of prostate tumorigenesis, and this reduction can result in compromised differentiation and a heightened risk of cancer [29]. A thorough functional analysis revealed that the NKX3.1–G9a–UTY transcriptional regulatory network plays a crucial role in prostate differentiation. Within this network, NKX3.1 engages in a physical interaction with the histone methyltransferase G9a (EHMT2), which is recognised as a chromatin modifier that plays a crucial role in regulating H3K9 methylation and differentiation programs [30] (Figure 2). Chromatin immunoprecipitation assays revealed that NKX3.1 directly interacts with the UTY promoter and facilitates the recruitment of G9a, leading to the transcriptional activation of UTY. In contrast, control genes like EDEM2 do not exhibit this G9a-dependent regulation. Experiments involving functional depletion have further confirmed that UTY plays a crucial role as a mediator in the differentiation of prostate cells driven by NKX3.1 [28]. The knockdown of UTY in human prostate epithelial cells (RWPE1 cell lines) expressing NKX3.1 resulted in the loss of induction of luminal differentiation markers such as AR, FOXA1, PSA, TMPRSS2, and HOXB13. In contrast, the control knockdown of EDEM2 did not produce any similar effects [29]. The findings collectively suggest a model in which the disruption of the NKX3.1–G9a–UTY axis undermines the fidelity of prostate lineage, making prostate epithelial cells more susceptible to malignant transformation [30].

### 2.2. Head and Neck Cancer

**Figure 3:**
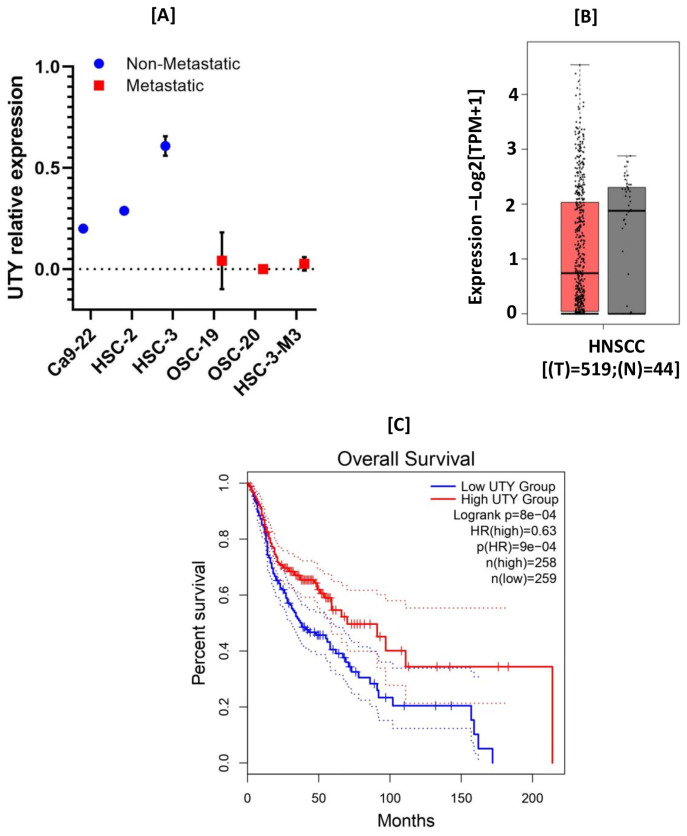
Expression of UTY in HNSCC: UTY expression in head and neck squamous cell carcinoma (HNSCC) tissues. (**A**) Subgroup analyses depicting UTY expression across the indicated non-metastatic (blue) and metastatic (red) cancer cell lines (as mentioned in the figure). (**B**) Box plot showing normalized UTY expression levels (log_2_[TPM + 1]) in tumour (T) (n = 519) (red) and normal (N) (n = 44) (grey) tissues. (**C**) Analysis of the survival plot revealed that the loss of UTY expression in patients is significantly associated with poor prognosis (*p* = 8 × 10^−4^). Data are presented as median (interquartile range). Statistical significance was set at *p* < 0.05. (**A**,**C**) (http://gepia2.cancer-pku.cn/ 28 March 2026).

In the context of head and neck malignancies, the role of UTY remains largely unexplored, though the broader dysregulation of histone-modifying enzymes, such as the NSD family of methyltransferases, is well-documented in head and neck squamous cell carcinoma pathogenesis [31]. Interestingly, our lab data revealed substantial UTY downregulation in both metastatic and non-metastatic oral cancer cell lines (Figure 3A). Ectopic UTY overexpression in these cells markedly suppressed proliferation and invasion (preliminary data). These findings warrant further validation, such as CRISPR-based UTY knockouts in female models (either in vitro or in vivo) to mimic loss of Y chromosome (LOY). Reinforcing this, in silico interrogation of public head and neck tumor datasets indicated progressively lower UTY expression with advancing tumor stages, alongside a strong link between UTY loss and worse patient prognosis (Figure 3B). However, specific investigations on the role of UTY in oral cancer is limited, but the functional significance of its paralog UTX has been characterized in oral tongue squamous cell carcinoma, where overexpression correlates with aggressive tumor progression and poor clinical outcomes [32]. However, analysis of TCGA data indicates that the loss of UTY expression in patients with HNSCC is significantly correlated with poor prognosis (Figure 3C). Given that UTX and UTY share high sequence homology and structural domains, it is plausible that UTY may similarly influence oncogenic pathways in head and neck tumors through its capacity to scaffold chromatin-modifying complexes, although direct empirical evidence is currently lacking.

### 2.3. Urothelial Carcinoma

In urothelial carcinoma, loss-of-function mutations in UTX, an H3K27 histone demethylase that antagonises EZH2 activity, have been reported in bladder cancer, suggesting that the absence of this catalytic function may shift the epigenetic balance toward a more oncogenic state [33]. Given that UTY is a paralog of UTX, its functional impact in urothelial carcinoma may differ significantly from the tumour-suppressive effects attributed to UTX, potentially acting through structural interactions within chromatin-modifying complexes rather than direct enzymatic regulation of H3K27me3 [34,35]. This structural capacity implies that UTY may function as a scaffold protein within chromatin remodelling assemblies, potentially influencing the recruitment or stability of transcriptional co-regulators that govern urothelial differentiation and proliferation [36]. Notably, copy number variations in the UTY gene have been reported in male patients afflicted with urothelial bladder cancer, suggesting that genomic alterations affecting this locus may contribute to disease pathogenesis [37]. Furthermore, the sex-specific expression patterns of UTY, given its location on the Y chromosome, may introduce distinct epigenetic vulnerabilities in male urothelial tumors that are not present in female counterparts, potentially influencing the observed disparities in cancer incidence and progression between sexes [15] (Figure 2).

### 2.4. Haematological Cancer

**Figure 4:**
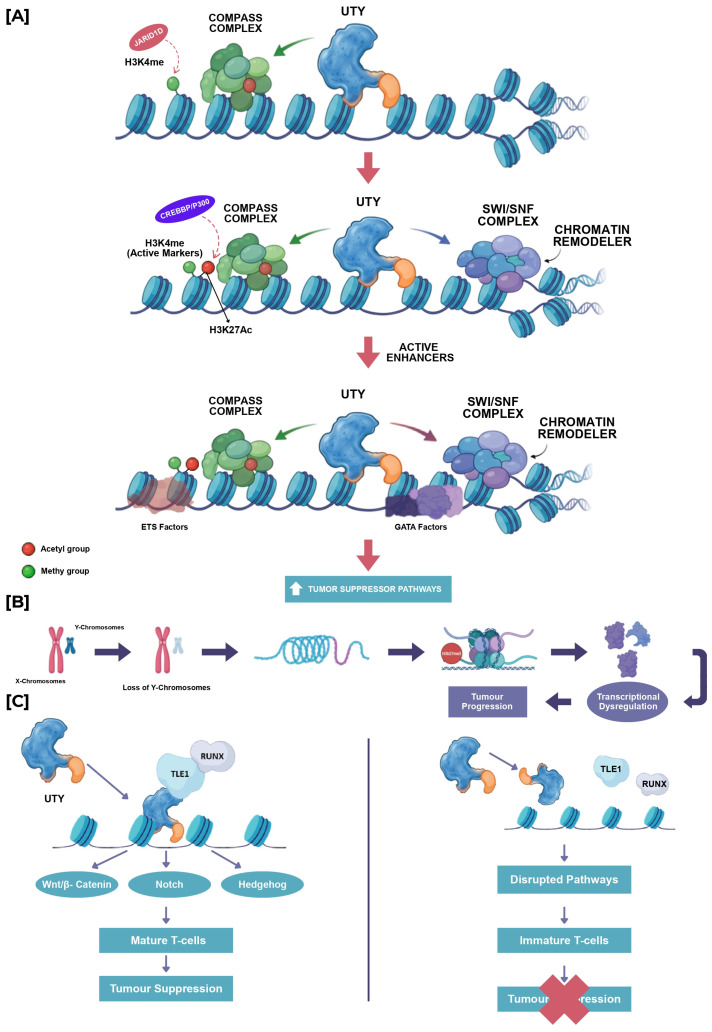
UTY-Regulated Epigenetic Remodeling and Its Role in Transcriptional Control and Tumour Suppression. (**A**) Schematic representation of UTY-mediated chromatin remodeling. UTY interacts with the COMPASS and SWI/SNF chromatin remodeling complexes. These complexes recognise the active histone marks, such as H3K4me (JARID1D-histone lysine 4 demethylase) and H3K27Ac (via CREBBP/p300-histone lysine 27 acetyl transferase). These modifications promote the formation of active enhancers and facilitate the binding of transcription factors, including GATA factors, thereby activating tumor suppressor pathways. (**B**) Overview of the consequences of epigenetic dysregulation. Disruption of the chromatin structure and alterations in histone modifications result in transcriptional dysregulation, thereby facilitating tumor progression. (**C**) Functional impact of UTY on signaling pathways and cellular differentiation. Under normal physiological conditions, UTY interacts with cofactors, such as TLE1 and RUNX, to regulate essential signaling pathways, including Wnt/β-catenin, Notch, and Hedgehog. This interaction facilitates the differentiation of mature T-cells and contributes to tumor suppression. In contrast, loss or dysfunction of UTY results in disrupted signaling pathways, impaired transcriptional regulation, immature T-cell states, and enhanced tumour progression.

In the context of haematological malignancies, the functional interplay between UTY and its paralog UTX has been investigated to determine whether UTY can compensate for the tumor-suppressive activity of UTX during tumorigenesis. Although UTY was previously considered devoid of demethylase activity, recent studies have shown that it can partially compensate for UTX function in knockout models, suggesting a potential role in maintaining epigenetic homeostasis [19,38]. However, UTY possesses markedly lower demethylase activity compared to UTX due to point substitutions affecting substrate binding, which limits its ability to directly reverse H3K27me3 modifications [22]. Despite this catalytic deficiency, functional studies suggest that UTY may retain the capacity to suppress myeloid leukemogenesis, as evidenced by studies showing that CRISPR-Cas9-mediated knockout of *Uty* in *Utx*-deficient mice increases hematopoietic stem cell self-renewal and recapitulates the leukemic phenotype observed in *Utx*-null females [22] (Figure 2). This finding is further supported by genomic analyses of human cancer cell lines, which reveal that concomitant loss of UTX and UTY is a frequent occurrence in male malignancies, including hematopoietic and solid organ cancers, where UTY microdeletions or mutations often accompany UTX inactivation [22]. Such concurrent inactivation implies that UTY may function as a haploinsufficient tumor suppressor in male cells, where its loss synergizes with UTX deficiency to promote oncogenic transformation through the disruption of chromatin regulatory networks [19,22]. Mechanistically, this tumor-suppressive capacity appears to operate through noncatalytic means, as global genomic profiling indicates that *Utx* loss results in significant alterations in chromatin accessibility, and H3K27Ac levels rather than widespread changes in H3K27me3 [22]. Specifically, UTX-mediated tumor suppression involves the inverse regulation of ETS and GATA transcriptional programs, a function that is shared with its catalytically inactive paralog UTY [22] (Figure 4A). This functional redundancy suggests that UTY can maintain critical chromatin regulatory networks and suppress leukemogenesis independently of lysine demethylase activity, a finding supported by mouse models where the presence of *Uty* or overexpression of catalytically inactive *Utx* mutants rescued developmental and preleukemic phenotypes associated with *Utx* deficiency [39].

Acute myeloid leukaemia (AML): In acute myeloid leukaemia, the role of UTY in the progression of the disease is significantly influenced by its loss associated with loss of the Y chromosome, a frequent somatic occurrence in aging men [40]. This event is closely related to clonal hematopoiesis, genomic instability, and an elevated risk of leukaemia [41]. The presence of UTY on the Y chromosome leads to a total absence of UTY gene expression in hematopoietic progenitor and stem cells due to the occurrence of LOY. In terms of its mechanism, UTY primarily functions in a non-enzymatic capacity within the process of hematopoiesis. UTY interacts with the transcriptional corepressor TLE1, and is brought to chromatin by RUNX1, which serves as a key regulator in the process of hematopoietic differentiation. In this intricate process, UTY may play a role in the suppression of RUNX1 target genes, thereby facilitating appropriate myeloid differentiation. The absence of UTY disrupts this repressive pathway, resulting in abnormal RUNX1 activity, hindered differentiation, and the buildup of immature myeloid progenitors, which is a characteristic feature of AML [42] (Figure 4C). Research in genomics indicates that the loss of UTY often coincides with mutations or deficiencies in UTX in male AML, leading to a significantly destabilized transcriptional landscape. At the chromatin level, the combined deficiency of UTY and UTX leads to changes in enhancer landscapes, resulting in dysregulation of H3K27ac and H3K4me1. This situation is accompanied by the inappropriate activation of oncogenic ETS-driven programs, as well as the repression of tumor-suppressive GATA pathways [22] (Figure 4A). Significantly, these defects can be remedied by enzymatically inactive UTX or UTY, indicating that the leukemia-suppressive role of UTY operates independently of histone demethylation (Figure 4C). Collectively, these findings suggest that UTY functions as a non-catalytic chromatin regulator. Its absence leads to the expansion and irregular self-renewal of hematopoietic progenitors, which in turn supports the onset and development of AML.

T-cell acute lymphoblastic leukemia (T-ALL): T-ALL exhibits a notable sex bias among hematological malignancies, characterized by a significant predominance in males. This phenomenon has been partially linked to variations in the gene dosage of UTX and UTY [22]. In females, two functional UTX alleles are preserved as a result of their escape from X-chromosome inactivation, while males depend on one UTX copy in conjunction with UTY. In this context, UTY functions as the only paralog that can partially offset the loss of UTX [43]. UTY’s role in T-ALL is primarily non-enzymatic and centres on maintaining chromatin organization and enhancer integrity at key developmental loci. Through its TPR domain, UTY assembles transcriptional repression complexes with TLE1 and RUNX1, which play essential roles in regulating T-cell receptor signaling, lineage commitment, and maturation. Loss of *UTY* disrupts RUNX1-mediated repression, leading to defective T-cell differentiation and persistence of immature or aberrantly programmed T-cell populations that are particularly susceptible to oncogenic transformation [22] (Figure 4C). When combined with UTX mutation or insufficiency, UTY loss results in profound disruption of enhancer landscapes, deregulated NOTCH1-associated transcriptional programs, and sustained self-renewal of leukemic T-cell progenitors [44]. Thus, UTY acts as a sex-specific modifier of disease penetrance and severity in T-ALL, mitigating oncogenic consequences of UTX loss in males and exacerbating leukemic transformation when absent [44].

### 2.5. Renal Cancer

In the context of renal cancer, especially clear cell renal cell carcinoma (ccRCC), the gene UTY has been associated with the recurrent somatic LOY and the subsequent transcriptional downregulation of Y-linked epigenetic regulators. Extensive genomic studies have shown that loss of the Y chromosome is among the most common chromosomal abnormalities observed in male ccRCC, with an occurrence rate of about 36–43% in tumours (Table 1) [45]. This anomaly is specific to tumors and differs from the age-related mosaic loss of the Y chromosome observed in peripheral blood [46]. Transcriptomic profiling demonstrated that UTY is among the Y-linked genes significantly downregulated in tumours harbouring somatic LOY, with expression levels inversely correlated with the proportion of tumor cells lacking chromosome Y. Notably, UTX is recurrently mutated or deleted in ccRCC and is recognized as a key tumour suppressor involved in chromatin remodelling and epigenetic regulation [47] (Figure 2). The study further highlighted that UTY loss in male tumours parallels UTX inactivation in female tumours, suggesting a sex-specific tumour-suppressive role for UTY in renal cancer. Although direct functional assays of UTY in renal cancer were limited, functional studies of another LOY-associated Y-linked demethylase, KDM5D, demonstrated that its ectopic expression significantly reduced the viability of LOY-positive renal cancer cell lines, supporting the broader concept that Y-linked epigenetic regulators act as tumor suppressors in ccRCC [48,49]. Taken together, these findings suggest that downregulation of UTY through somatic LOY contributes to renal cancer pathogenesis by reinforcing epigenetic instability and disrupting chromatin-based regulatory networks that normally constrain tumor development (Figure 4).

### 2.6. Breast Cancer

Given the limited direct evidence for UTY in breast cancer, the current understanding is largely extrapolated from studies on its paralog UTX. In breast cancer subtypes, the EZH2-H3K27me3 axis has been shown to drive metastatic progression, with EZH2 expression correlating with KRT14 levels in basal-like tumours and peritoneal metastasis. Although, EZH2-independent mechanisms involving demethylases like UTX may also contribute to H3K27me3 regulation in other metastatic sites [50]. High expression levels of EZH2 are considered an indicator of aggressive breast cancer, and is involved in tumour initiation, progression, metastasis, and stem cell regulation [51]. EZH2 is frequently elevated and facilitates invasive tumour growth, suggesting that the interplay between UTY and the PRC2 complex may similarly influence the epigenetic landscape and therapeutic response of these malignancies [52]. The Luminal B subtype, characterized by particularly poor survival outcomes due to a lack of viable therapeutic options, highlights the necessity of defining how distinct transcriptional networks are uniquely modulated by genetic and epigenetic mechanisms [15,51,53]. Tri-methylation of lysine 27 on histone 3 by the methyltransferase EZH2, as a part of the polycomb repressive complex 2, is an important mechanism of gene silencing [51].

**Table 1:** **Frequency and nature of UTY-associated genomic alterations across different cancer types:** Quantitative data from TCGA and related genomic studies, highlighting the predominance of loss of chromosome Y (LOY) as the primary mechanism of UTY loss in cancer, in contrast to the higher mutation frequency observed for its paralog UTX (KDM6A).

Cancer Type	UTY- Related Alteration	Frequency (%)	Key Observation	Evidence Type	References
Bladder cancer (UTX comparison)	UTX mutation (contrast with UTY)	~25–30%	UTX frequently mutated, whereas UTY alterations are primarily due to LOY rather than mutation	Genomic studies	[33,35]
Acute myeloid leukaemia (UTX comparison)	UTX mutation	~8–10%	Reinforces differential contribution of UTX vs. UTY in leukemogenesis	Genomic studies	[35]
Renal cell carcinoma (UTX comparison)	UTX mutation	~10–15%	Highlights functional divergence between UTX (mutational inactivation) and UTY (dosage loss via LOY)	Genomic studies	[35,47]
Acute myeloid leukaemia (AML)	LOY	~10–15%	Associated with clonal hematopoiesis, genomic instability, and poor prognosis	Clinical cohorts/Genomic analyses	[40,54]
Aging male population (baseline comparison)	Mosaic LOY	~10–20%	Age-related somatic event predisposing to hematological malignancies	Population studies	[40,54]
Clear cell renal cell carcinoma (ccRCC)	Loss of chromosome Y (LOY)	36–43%	Frequent chromosomal loss leading to downregulation of UTY and other Y-linked epigenetic regulators; associated with tumour progression	TCGA/Genomic studies	[45,47]
Pan-cancer	UTY mutation	Rare	UTY mutations are infrequent; functional loss is primarily driven by LOY	Genomic studies	[47,55]
Pan-cancer (lung, bladder, colorectal, etc.)	LOY	20–40% (variable by cancer type)	One of the most common somatic alterations in male cancers; reflects loss of Y-linked gene dosage including UTY	TCGA/Pan-cancer analyses	[54,55]

### 2.7. Other Solid Tumors

In addition to its implications in bladder cancer and haematological malignancies, the loss of UTY is gaining recognition as a significant factor in a wide range of solid tumors [47,56], primarily due to its connection with LOY. Extensive epidemiological and genomic research indicates that LOY is prevalent in various cancers, including those of the lung, prostate, and colon. This phenomenon is linked to unfavourable outcomes, heightened genomic instability, and an increased risk of cancer-related mortality among men [54]. The loss of UTY results in the elimination of a crucial regulator of transcriptional balance [35]. UTY is part of a conserved group of chromatin regulators characterized by X–Y pairing, which also includes UTX, and plays a crucial role in the organization of chromatin and the regulation of gene expression. Despite UTY’s limited intrinsic demethylase activity, it plays a significant role in COMPASS-related chromatin regulatory networks, contributing to the equilibrium between transcriptionally active and repressive chromatin states [35]. The reduction in UTY levels leads to a decreased dosage of the KDM6 family in male cells, which promotes the accumulation of H3K27me3, inhibits the expression of genes associated with differentiation, and enhances cellular plasticity. Pan-cancer analyses of TCGA datasets have demonstrated that loss of chromosome Y (LOY) is one of the most common somatic alterations in male cancers, with frequencies ranging from 20% to 40% depending on tumour type (Table 1), including lung, bladder, and colorectal cancers. This recurrent loss leads to reduced expression of Y-linked genes such as UTY, supporting a dosage-dependent role in epigenetic regulation [54,55]. This observation aligns with the notion that UTY may function as a tumor suppressor, influencing chromatin organization instead of acting through catalytic mechanisms [43,55]. Beyond the direct effects on tumor cells, the loss of UTY may also facilitate the progression of solid tumors by influencing immune responses. The presence of LOY in hematopoietic cells has been associated with immune dysfunction, changes in T-cell responses, and a rise in cancer mortality. This indicates that a deficiency in UTY may hinder immune surveillance and promote tumour evasion [35,54]. Recent findings suggest that the loss of UTY driven by LOY may leads to common vulnerabilities and synthetic-lethal dependencies on X-linked paralogs like DDX3X and EIF1AX, which may present potential avenues for therapeutic intervention [55]. The findings collectively suggest a model in which UTY serves as a widely influential chromatin-associated tumour suppressor. Its loss appears to play a significant role in the development of cancer by leading to transcriptional dysregulation, hindered differentiation, and immune evasion across various types of cancer.

## 3. Discussion

The collective findings from haematological, prostate, urothelial, renal, head and neck cancer malignancies underscore a paradigm shift in understanding UTY’s contribution to cancer biology, moving beyond its historical classification as a catalytically inactive relic to recognizing its essential role as a structural component of chromatin regulatory machinery. Rather than serving as a redundant evolutionary vestige, UTY exerts significant influence on oncogenic transcriptional programs through its capacity to scaffold critical co-activator complexes and modulate enhancer landscapes across diverse tissue types. This scaffolding function is particularly critical in the context of the COMPASS complex, where UTY interacts with KMT2C/D to maintain H3K4 mono-methylation and facilitate the recruitment of ATP-dependent chromatin remodelers such as SMARCA4 and CHD4, thereby preserving the epigenetic landscape required for normal cellular differentiation and suppressing malignant transformation [22,57,58,59]. Although, the therapeutic implications of targeting UTY’s noncatalytic functions are underscored by evidence that UTX-mediated tumor suppression operates through inverse regulation of ETS and GATA transcriptional programs, a mechanism that can be recapitulated by catalytically inactive mutants [22,58,60]. This mechanistic insight suggests that pharmacological strategies aimed at restoring or mimicking UTY’s scaffolding activity, rather than inhibiting enzymatic function, may offer a novel therapeutic avenue for malignancies characterized by UTY deficiency [22]. Ultimately, recognizing UTY as a critical structural effector within chromatin regulatory networks provides a refined framework for understanding sex-specific disparities in cancer susceptibility and informs the development of precision therapies targeting the epigenetic dependencies of UTX/UTY-deficient tumors [58]. Notably, UTY represents a critical noncatalytic component of the chromatin regulatory machinery, functioning as a structural scaffold that maintains enhancer activity and suppresses oncogenic transcriptional programs through its integration into COMPASS and SWI/SNF complexes. Disruption of this recruitment mechanism, as observed in models of SMARCA4 deficiency, leads to the closure of chromatin regions and loss of accessibility at GATA-bound enhancer sites, highlighting the functional interdependence between COMPASS-mediated histone modifications and SWI/SNF-dependent chromatin remodelling (Figure 4). Such interdependence reveals that the integrity of the epigenetic landscape relies on the cooperative action of histone modifiers and ATP-dependent remodels, where the loss of one component can precipitate widespread transcriptional dysregulation [8,22,58]. This vulnerability is further illustrated by the observation that SMARCA4-mutant cells exhibit a strong dependency on KDM6A/UTX and KDM6B/JMJD3, such that inhibition of these histone demethylases compromises cell viability and demonstrates anti-tumour effects in preclinical models. These findings collectively highlight the potential for exploiting synthetic lethal interactions in epigenetically dysregulated cancers, where the disruption of chromatin homeostasis creates unique therapeutic vulnerabilities that can be targeted to restore normal gene expression patterns. Therapeutic strategies may therefore focus on the development of small molecules or degraders that specifically modulate the assembly or stability of these chromatin regulatory complexes to correct aberrant transcriptional programs in malignancies with compromised UTY or UTX function.

## 4. Future Perspective

Future cancer research should prioritize identifying the exact structural features underlying UTY’s scaffolding role and uncovering synthetic lethal vulnerabilities for therapeutic targeting in tumours lacking UTX/UTY. Such efforts may ultimately lead to the development of targeted therapies that restore epigenetic balance and improve outcomes for patients with these aggressive cancers. Specifically, investigating the dynamic composition of SWI/SNF complexes in haematological malignancies could reveal novel synthetic lethality relationships, as residual complexes may gain oncogenic functions upon subunit inactivation that can be pharmacologically targeted. For instance, the synthetic lethal relationship between PRC2 inhibition and SMARCB1 loss represents one of the most actionable dependencies currently targetable with inhibitory compounds against the core subunits EZH2 and EED. Ongoing clinical trials of EZH2 inhibitors, such as tazemetostat (an FDA-approved EZH2 inhibitor), provide strong precedent for PRC2-targeted therapies in SMARCB1-deficient cancers like epithelioid sarcoma (e.g., Phase 2 TRITON trial, NCT02601950, showing ~15% objective response rates). These successes underscore the potential for analogous PRC2 inhibition in UTY-deficient tumors exploiting similar synthetic lethal vulnerabilities. Nonetheless, key hurdles persist, i.e., the inherent druggability challenges of chromatin scaffold proteins like UTY or SMARCA4, which lack deep enzymatic pockets; risks of off-target effects on paralogous pathways (e.g., EZH1 compensation or non-specific H3K27me3 disruption).This conceptual framework for combinatorial therapy is supported by the growing understanding of crosstalk mechanisms between Polycomb and other epigenetic modifications, which provides a rationale for targeting the dysregulated epigenetic landscape characteristic of cancer.

## Data Availability

No new data were created or analyzed in this study. Data sharing is not applicable to this article.

## Abbreviations

KDM6A	Lysine (K) Demethylase 6A
KDM6B	Lysine (K) Demethylase 6B
KDM6C	Lysine (K) Demethylase 6C
UTY	Ubiquitously Transcribed Tetratricopeptide Repeat Containing, Y-Linked
UTX	Ubiquitously Transcribed Tetratricopeptide Repeat, X-Linked
SWI/SNF	SWItch/Sucrose Non-Fermentable
COMPASS	Complex Of Proteins Associated with Set1
PRC2	Polycomb Repressive Complex 2
EZH2	Enhancer of Zeste Homolog 2
EED	Embryonic Ectoderm Development
SMARCB1	SWI/SNF Related, Matrix Associated, Actin Dependent Regulator of Chromatin, Subfamily B, Member 1
SMARCA4	SWI/SNF Related, Matrix Associated, Actin Dependent Regulator of Chromatin, Subfamily A, Member 4
TLE1	Transducin-Like Enhancer of Split 1
CHD4	Chromodomain Helicase DNA Binding Protein 4
ETS	E26 Transformation-Specific
NKX3.1	NK3 Homeobox 1
RUNX1	Runt-Related Transcription Factor 1
GATA	Named After DNA Binding Motif “GATA” (Guanine-Adenine-Thymine-Adenine)
LOY	Loss of Chromosome Y
JmjC	Jumonji C
CREBBP	CREB-binding Protein
TPR	Tetratricopeptide Repeat Domain
T-ALL	T-cell Acute Lymphoblastic Leukemia
AML	Acute Myeloid Leukaemia
ccRCC	Clear Cell Renal Cell Carcinoma
